# Agarose Gel-Templating Synthesis of a 3D Wrinkled Graphene Architecture for Enhanced Supercapacitor Performance

**DOI:** 10.3390/mi13071113

**Published:** 2022-07-15

**Authors:** Junhyeop Shin, Jong-Kwon Park, Geon Woo Kim, Inho Nam, Soomin Park

**Affiliations:** 1 School of Chemical Engineering and Materials Science, Department of Intelligent Energy and Industry, Department of Advanced Materials Engineering, Chung-Ang University, Seoul 06974, Korea; wnsguqdl123@cau.ac.kr (J.S.); godwls89@cau.ac.kr (J.-K.P.); 2 School of Energy, Materials and Chemical Engineering, Korea University of Technology and Education, Cheonan 31253, Korea; gunwoo0428@koreatech.ac.kr

**Keywords:** graphene, reduced graphene oxide, agarose gel, soft template, supercapacitor, electrical double-layer capacitor

## Abstract

The increasing use of rapidly fluctuating renewable energy sources, such as sunlight, has necessitated the use of supercapacitors, which are a type of energy storage system with high power. Chemically exfoliated graphene oxide (GO) is a representative starting material in the fabrication of supercapacitor electrodes based on reduced GO (rGO). However, the restacking of rGO sheets driven by π–π stacking interactions leads to a significant decrease in the electrochemically active surface area, leading to a loss of energy density. Here, to effectively inhibit restacking and construct a three-dimensional wrinkled structure of rGO (3DWG), we propose an agarose gel-templating method that uses agarose gel as a soft and removable template. The 3DWG, prepared via the sequential steps of gelation, freeze-drying, and calcination, exhibits a macroporous 3D structure and 5.5-fold higher specific capacitance than that of rGO restacked without the agarose template. Further, we demonstrate a “gel-stamping” method to fabricate thin-line patterned 3DWG, which involves the gelation of the GO–agarose gel within micrometer-sized channels of a customized polydimethylsiloxane (PDMS) mold. As an easy and low-cost manufacturing process, the proposed agarose gel templating method could provide a promising strategy for the 3D structuring of rGO.

## 1. Introduction

It is extremely important to use renewable energy sources to achieve the goal of carbon neutrality. The power output of most renewable energy sources, such as sunlight and wind, exhibits natural fluctuations, necessitating the use of energy storage devices to achieve a balance between energy supply and demand [1]. Among the various types of energy storage devices (e.g., rechargeable battery [2], supercapacitor [3]), supercapacitors are especially significant because they offer rapid charge–discharge ability with low energy losses, as well as a long cycling life and excellent power density [4]. However, their relatively low energy density is a critical limitation that has hindered the broad application of supercapacitor systems [5]. Therefore, extensive research has been devoted toward increasing the energy density of electrode materials in supercapacitors.

The electrical double-layer capacitor (EDLC) is the archetypal supercapacitor that stores electrostatic charges on a biased electrode surface. Carbon allotropes are the most commonly used electrode materials in EDLCs, and their electrochemically active surface area and electrical conductance primarily determine the energy density of EDLCs. Although porous activated carbon is widely used in commercial EDLC systems, graphene has attracted attention as a next-generation electrode material owing to its large surface area and high electrical conductance. Techniques for the chemical exfoliation of graphene sheets from graphite have been optimized, with current techniques involving the use of the improved Hummer’s method [6]. This process involves the oxidation of graphene sheets and subsequent exfoliation by weakening the π–π stacking interactions. The produced graphene oxide (GO) sheets need to be chemically reduced to restore their high electrical conductance, yielding reduced GO (rGO). During the wet chemical reduction of GO, the rGO sheets are restacked by van der Waals forces, and their electrochemically active surface area significantly decreases. Therefore, the inhibition of rGO restacking and construction of porous rGO networks without the loss of electrical conductance has attracted growing scientific and industrial attention to the energy storage applications of rGO [7,8,9,10,11,12,13,14].

Agarose is a linear polymer derived from red seaweed and is composed of repeating units of a disaccharide comprising D-galactose and 3,6-anhydro-L-galactopyranose [15]. Simple heat-up (to ~90–95 °C) and subsequent cooling (to room temperature) in water form a three-dimensional (3D) interconnected porous structure of gelated agarose. Owing to its water solubility, tunable porosity, scalable production, and environmental friendliness, the soft agarose gel template has been successfully utilized to construct 3D porous structures of metals and metal oxides in our previous research [16,17,18,19]. Here, we demonstrate a facile method to fabricate a 3D wrinkled structure of rGO (3DWG) using the soft agarose gel template. As an EDLC electrode material, the electrochemically active surface area and energy density of the prepared 3DWG were higher than those of rGO restacked without the agarose gel template. Further, inspired by the process of wax seal stamping, we proposed an effective way to fabricate a micro-device with thin-lined patterns of the 3DWG, which could be useful for fabricating an all-solid-state transparent supercapacitor chip [20,21,22].

## 2. Materials and Methods

### 2.1. Synthesis of the 3DWG

To chemically exfoliate graphite, graphite oxides were prepared using the previously reported improved Hummer’s method [6]. Yellow-colored graphite oxide (6 g) and agarose powder (2.5 g, Sigma Aldrich, Burlington, VT, USA) were dispersed in 100 mL of deionized water to form Solution 1, which was ultrasonicated for 1 h to exfoliate graphite oxide, yielding a mixed solution of GO and agarose. The mixed solution was heated in a microwave oven for 1 min to completely dissolve the agarose powder, forming Solution 2, which was then poured onto a petri dish for cooling at room temperature. After cooling for 6 h, the GO-containing agarose gel was obtained, which was cut into pieces of the desired size and freeze-dried (Bondiro, Ilshinlab, Yangju, Korea) to sublimate the water inside the gel pores. The composite comprising the freeze-dried GO and agarose was subsequently calcined in air at 500 °C for 6 h to remove the agarose template and completely reduce GO to rGO.

### 2.2. Characterization

The structural information of 3DWG was characterized by X-ray diffractometer (XRD, M18XHF-SRA diffractometer, MAC Science, Yokohama, Japan) and Raman spectroscopy (RM-1000, Renishaw, Gloucestershire, UK). The surface morphology of the prepared rGO samples was characterized by scanning electron microscopy (SEM, SUPRA 55VP, Carl Zeiss, Oberkochen, Germany). Bright-field transmission electron microscopy (TEM) images were obtained using a high-resolution TEM (HR-TEM) apparatus (JEM-3010, JEOL, Tokyo, Japan). For the electrochemical measurements, cyclic voltammetry (*CV*) was performed in a standard three-electrode configuration (IviumStat electrochemical analyzer, Ivium Technology, Eindhoven, The Netherlands) with an aqueous 1.0 M Na_2_SO_4_ solution as the electrolyte. A saturated Ag/AgCl electrode and Pt plate were used as the reference and counter electrodes, respectively. The working electrodes were prepared by coating 80 wt% rGO materials (3DWG and restacked rGO), 10 wt% polytetrafluoroethylene (PTFE) as a binder, and 10 wt% Ketjenblack on stainless steel (SUS) mesh.

### 2.3. Calculations

The specific capacitance (F·g^−1^) was calculated from the cyclic voltammograms (CV) according to the following equation:

$$C=\frac{ln_{CV}}{v\times \Delta V\times m}$$

where $ln_{CV}$ is the integral area for the discharged CV curve (IV), v is the sweep rate (V·s^−^^1^), ΔV is the potential window (*V*), and m is the mass of the electrode materials (g).

The specific power (*P*, W·kg^−^^1^) at a certain scan rate v (V·s^−^^1^) was calculated by integrating the discharged area (IV) of the CV curves as follows:

$$P=\int_{V_{1}}^{V_{2}}\frac{I}{m}dv$$

where V_1_ and V_2_ are 0.0 and 0.8 V.

The specific energy (*W*, W·kg^−1^) was obtained using the following equation:

$$W=\frac{V_{2}-V_{1}}{v\times 3600}\times \int_{V_{1}}^{V_{2}}\frac{I}{m}dv$$

### 2.4. Fabrication of Thin-Lined Patterns of the 3DWG

A photoresist layer was spin-coated and developed on a Si wafer using a photomask (MA-6, Karl-Suss, Garching, Germany). After etching with hydrofluoric acid (etching depth: ~10 µm), the residual photoresist was removed using acetone, and then by piranha solution (H_2_O_2_:H_2_SO_4_ = 1:3) treatment for complete cleaning. A polydimethylsiloxane (PDMS) base and curing agent (Sylgard 184, Dow Corning, Midland, TX, USA) were mixed in a 7:3 (*w*/*w*) ratio and poured onto the etched Si wafer. The prepared PDMS mold was obtained after de-bubbling under a vacuum and subsequent backing at 100 °C for 2 h. The viscous Solution 2 (refer to Section 2.1) was poured onto the piranha-cleaned glass substrate, and the prepared PDMS mold was pressed down firmly, similar to wax seal stamping. After the gelation of agarose within the PDMS channel at room temperature, the PDMS mold was peeled off, leaving a thin-lined pattern of the GO-containing agarose gel on the glass substrate. Freeze-drying followed by calcination finally produced a thin-lined pattern of the 3DWG without the agarose template.

## 3. Results

**Scheme 1:**
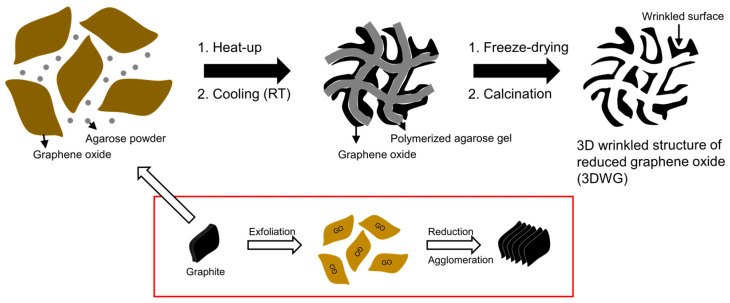
Schematic of the fabrication process for the 3D wrinkled structure of rGO (3DWG). The red box highlights the process for the formation of restacked rGO without the agarose gel template.

Scheme 1 shows the overall process of 3DWG fabrication. The hydrophilic nature of both GO and agarose facilitates intimate contact between these materials, which maintain a well-mixed state during the rapid polymerization of the agarose gel at room temperature. The 3D interconnected structure of the agarose gel traps the GO within the pores and hinders restacking upon reduction to rGO. The freeze-drying process sublimates the water present in the agarose gel while preserving its original 3D structure. After calcination in air, the dried agarose template is completely removed and the remaining GO sheets are reduced to rGO with a 3D wrinkled structure. The key chemical features, including hydrophilicity and facile polymerization (occurring during heat-up and cool-down, respectively), render the agarose gel usable for the 3D structuring of graphene sheets. Without the agarose gel template, the GO sheets are restacked upon reduction to rGO, which is highly detrimental to the energy density of EDLCs because of the sharp decrease in the electrochemically active surface area.

**Figure 1:**
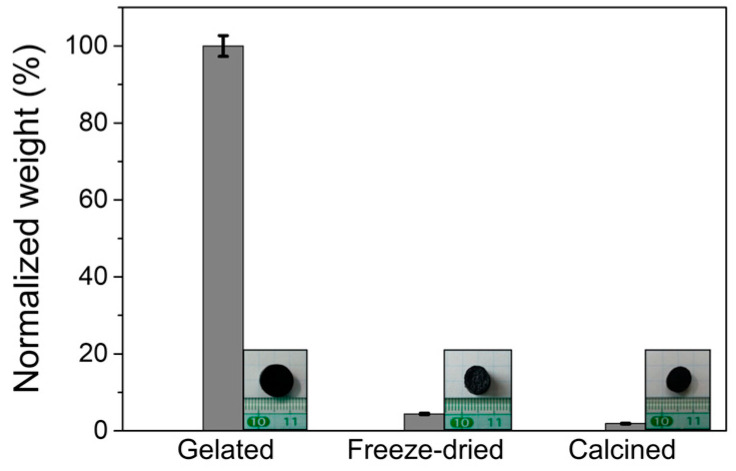
Normalized weight loss of the GO–agarose gel composite after freeze-drying and subsequent calcination. The insets indicate the corresponding samples, respectively.

To prepare the 3DWG, we cut out a cylindrical piece of the GO–agarose gel (1 cm diameter and 0.5 cm height). Figure 1 shows the normalized weights of the GO–agarose gel, freeze-dried GO–agarose gel, and calcined 3DWG. During the freeze-drying of the GO–agarose gel, the initial weight is reduced by 95.6%, presumably due to the sublimation of water in the agarose gel pores. Calcination causes the freeze-dried GO–agarose gel to lose 57% of its weight, meaning that the mass of GO–agarose decreased with the reduction. Based on the previous research, half of GO can remain as rGO at the calcination temperature (500 °C) [6]. On the other hand, agarose is almost eliminated at over 450 °C [16,23,24]. Therefore, the fabricated sample is almost composed of rGO, not agarose. This series of steps constitutes an easy and low-cost manufacturing process to obtain a freestanding 3D-structured graphene electrode.

**Figure 2:**
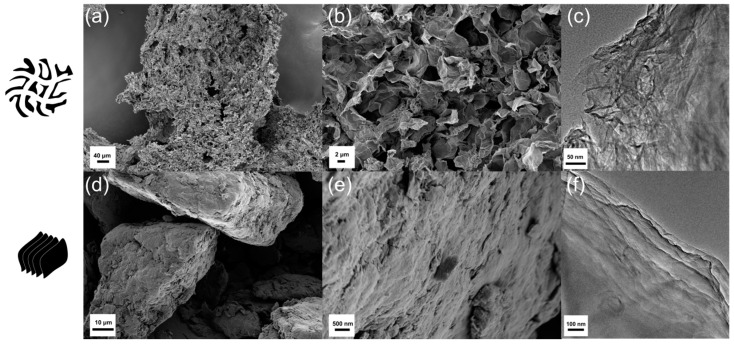
SEM images of (**a**,**b**) 3DWG and (**d**,**e**) restacked rGO without the agarose template. HR-TEM images of (**c**) 3DWG and (**f**) restacked rGO.

Figure 2a,b show the SEM images of the 3DWG at low and high magnifications, respectively. The observed porous structure of the 3DWG is replicated from the intrinsic porous nature of the agarose gel [16]. The 3D interconnected rGO structure appears to have a few micrometer-sized macropores where the (now-removed) water and agarose earlier existed. The TEM image (Figure 2c) confirms that the 3DWG surface is wrinkled, which can enable increased ion accessibility and provide a large electrochemically active surface area for EDLC applications. Conversely, an rGO sample prepared without the agarose gel template shows extensively restacked structures (Figure 2d,e). The aforementioned π–π interactions cause the rGO sheets to preferably form into multilayered stacked structures, as observed in the TEM image (Figure 2f). Such restacked rGO structures, as expected, would have a limited ion-accessible surface area and low energy density as an EDLC electrode material. Therefore, the 3DWG benefits from the high electrical conductivity of rGO, as well as the enhanced surface area, which are key characteristics to obtain both the required power and energy densities in EDLCs, respectively.

**Figure 3:**
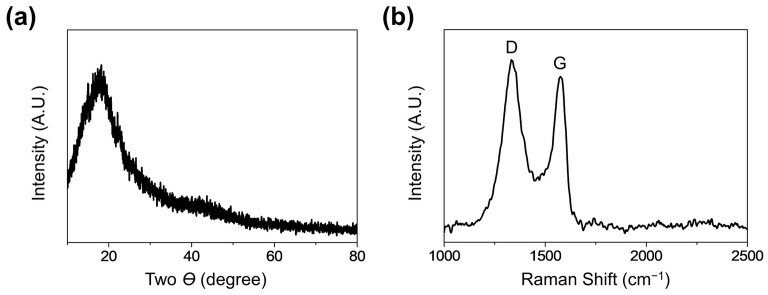
(**a**) X-ray diffraction and (**b**) Raman spectra of 3DWG.

To obtain structural information on 3DWG, we conducted X-ray diffraction and Raman characterization. The XRD data show a peak at ~20° corresponding to (002) plain (Figure 3a). The peak is located between peaks of the (002) plane of graphite (26.5°) and GO (9.3°), meaning that the spacing of 3DWG is decreased by GO reduction, however, it is still broad compared with bulk graphite. Based on Raman spectroscopy in Figure 3b, 3DWG shows two major peaks of the D and G band at 1326 cm^−1^ and 1575 cm^−1^. The *I_D_/I_G_* intensity ratio of 3DMG is estimated to be 1.13, which suggests the reduced average size of the sp_2_ domain upon reduction of the GO. It indicates the removal of hydroxyl, epoxy, and carbonyl groups. The value, 1.13, is smaller than other *I_D_/I_G_* intensity ratios for 3D graphene with chemical reduction, meaning that the 3DWG maintains the ideal graphitic nature relatively well [10,25,26].

**Figure 4:**
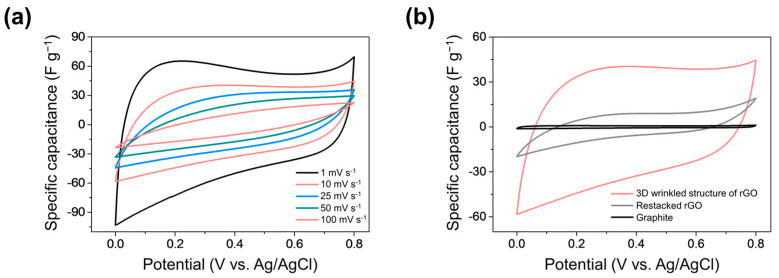
(**a**) Cyclic voltammograms of the 3DWG, restacked rGO, and graphite electrodes at a scan rate of 10 mV·s^−^^1^. (**b**) Cyclic voltammograms of the 3DWG electrode at various scan rates in the range of 1−100 mV·s^−^^1^.

To evaluate the electrochemical performance of the 3DWG electrode, we conducted *CV* measurements in a potential window of 0.0–0.8 V (vs. Ag/AgCl). These measurements were performed using a three-electrode system in a 1 M Na_2_SO_4_ aqueous electrolyte. Figure 4a shows the specific capacitances of the 3DWG as a function of the potential at scan rates of 1, 10, 25, 50, and 100 mV·s^−1^. The *CV* curves exhibit equivalent charge and discharge areas, indicative of excellent Coulombic efficiency and capacitive behaviors. The specific capacitance of the 3DWG is calculated to be 56.6 F·g^−1^ at a scan rate of 1 mV·s^−1^. To compare the performance of the 3DWG with that of the restacked rGO and graphite electrodes, the *CV* curves of the three electrodes at a scan rate of 10 mV·s^−1^ are shown in Figure 4b. Among all three electrodes, the 3DWG electrodes offer radically improved electrochemical performance compared to those of their counterparts. Specifically, the 3DWG electrode shows 5.5 and 43 times higher specific capacitances than rGO restacked without the agarose template and graphite, respectively. The electrochemically active surface area of the 3DWG is 270 m^2^·g^−1^, assuming that the intrinsic capacitance of graphene is 21 µF·cm^−2^ [27]. Considering the theoretical specific surface area of the graphene monolayer as 2620 m^2^·g^−1^ [28], the utilization rate of the graphene surface for the formation of the electrical double layer reaches 10.3%. For comparison, the utilization rates for restacked rGO and graphite are below 0.01%.

**Figure 5:**
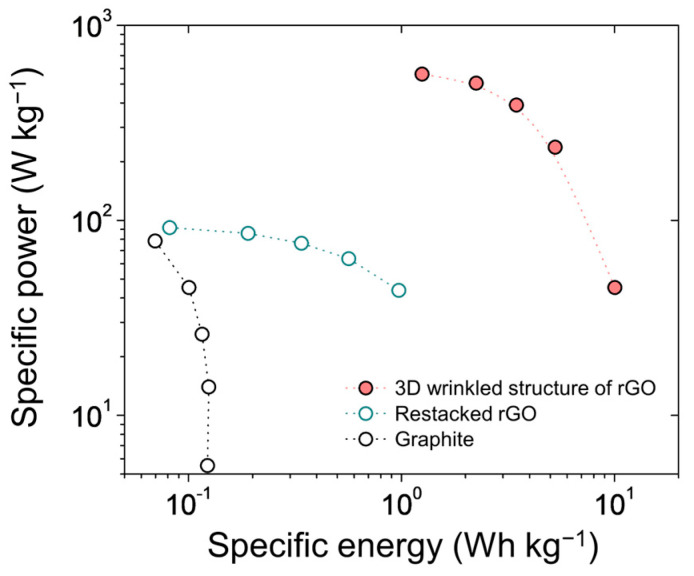
Specific energy and specific posswer of the 3DWG, restacked rGO, and graphite electrodes.

Figure 5 shows the specific energy and power calculated from the *CV* data (Figure 4). The 3DWG electrode shows a specific power range of 45–562 W·kg^−1^ and a specific energy range of 1.2–10 Wh·kg^−1^. In contrast, the maximum specific energies of restacked rGO and graphite are 0.97 Wh·kg^−1^ and 0.12 Wh·kg^−1^, respectively. The superior energy and power densities of the 3DWG electrode suggest that the 3D structuring of rGO considerably improves the electrochemically active surface area without the significant obstruction of the ion transport during charge and discharge. The rGO sheets can serve as conductive platforms for loading heterogeneous active materials. In fact, many studies have been conducted to synthesize metal oxide-decorated graphene sheets for supercapacitor and battery applications [29]. Although the specific energy and power of the 3DWG electrode could be further increased by the deposition of pseudocapacitive materials, such as MnO_x_ and RuO_x_, the current study focuses on a 3D structuring method for rGO.

**Scheme 2:**
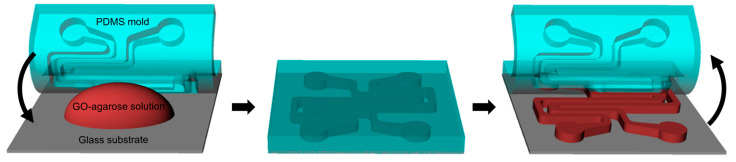
Schematic of the fabrication of thin-lined patterns of the 3DWG using a “gel-stamping” method.

The fabrication process of the 3DWG involves the use of a highly viscous GO–agarose solution (Solution 2, see Materials and Methods—Section 2.1). Similar to traditional glass art with fused silica, this pre-gelated solution containing dissolved agarose provides a window of time for creating the desired shape of the final freestanding 3DWG. As a proof of concept, we fabricated a thin-lined pattern of the 3DWG using an engraved PDMS mold with a few hundred of micrometer-sized wells [30]. Scheme 2 shows the overall process for the fabrication of thin-lined patterns of the 3DWG. The PDMS mold was prepared via the reverse replication of an etched Si wafer. The viscous GO–agarose solution was cast on a glass substrate, and the PDMS mold was then pressed onto the cast solution. The GO–agarose solution was trapped in the engraved channels of the PDMS mold, and the residual amount of the solution was squeezed out of the combined PDMS–glass. After gelation at room temperature, the PDMS mold was lifted off, leaving a thin-lined pattern of the GO–agarose gel. The hydrophobic surface of PDMS enables the retention of the hydrophilic GO–agarose gel on the glass substrate without attachment to the lifted PDMS. This series of processes is highly analogous to wax seal stamping; therefore, we named it as the “gel-stamping” method.

**Figure 6:**
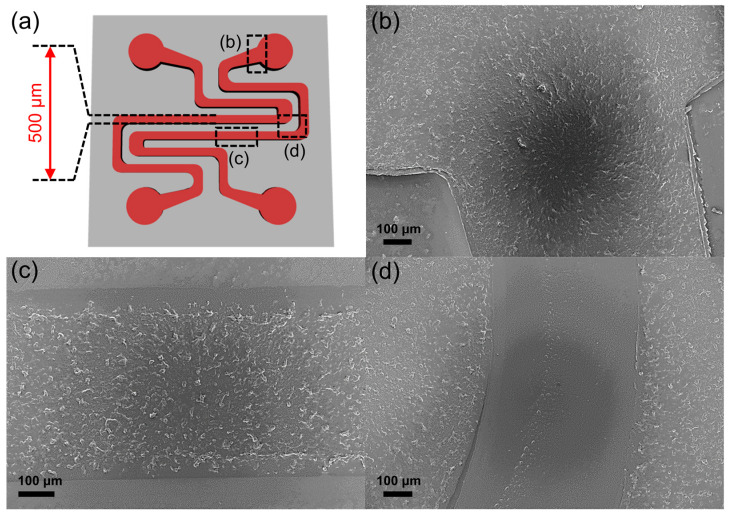
(**a**) Schematic of the thin-lined patterns of the 3DWG. Labeled areas denoted with dashed-line boxes represent areas for SEM analyses. (**b**–**d**) SEM images of thin-lined patterns of the 3DWG constructed on a glass substrate.

A PDMS mold with two engraved channels (500 µm in width and 100 µm in height) was used for the gel stamping of GO–agarose, which produced two parallel lines of the 3DWG. Figure 6a illustrates the thin-lined patterns of the 3DWG. Since the subsequent freeze-drying and calcination steps do not involve significant volumetric shrinkage (photographs in Figure 1), the produced thin-lined pattern of the 3DWG exhibits no breaks or defects. Figure 6b–d show SEM images of sections of the patterned 3DWG. The pattern successfully develops the 3D wrinkled structure with a clear edge and without any breaks or defects. Further, the PDMS mold is reusable.

Considerable efforts have been devoted to the fabrication of patterned graphene because such microscale energy storage units are especially important for integrating energy conversion devices (such as piezoelectric nanogenerators, solar cells, or thermoelectric cells) to build self-powered micromachine systems [31,32]. The proposed gel-stamping method utilizes the unique features of agarose, such as easy dissolution in water, gelation at room temperature, and formation of 3D interconnected structures even with acidic impurities (GO). Compared to the developed techniques, including laser-induced graphene and chemical vapor deposition [33,34,35], this agarose gel-stamping method has the distinct advantages of solution processability, scalability, and cost-effectiveness.

## 4. Conclusions

In this study, we proposed a method for the 3D structuring of rGO materials to increase their electrochemically active surface area and the energy density of the supercapacitor. As a soft template, the agarose gel possessed macropores and was hydrophilic, which provided a suitable environment for the structuring of rGO. The formation of a solution-processible GO–agarose gel, as well as its freeze-drying and calcination, illustrated the facile processability of the freestanding 3DWG electrode. Electrochemical measurements revealed that the specific capacitance of the 3DWG was 5.5 times higher than that of rGO restacked without the agarose template, implying that the 3DWG could be a useful platform for electrode materials combined with pseudocapacitive metal oxides. The most important advantage of the use of agarose is that we could easily manipulate the final shape of the freestanding 3DWG by using a customized container or mold for the gelation of GO–agarose. As a proof of concept, a thin-lined pattern of the 3DWG was successfully fabricated by using a PDMS mold, making our method promising for the fabrication of microchip supercapacitors. We believe that the agarose gel-templating method proposed herein will be of significance in expanding the use of graphene-based electrodes for energy storage systems, including supercapacitors and rechargeable batteries.
